# New records of amphipod crustaceans along the Israeli Mediterranean coast, including a rare Mediterranean endemic species, *Maera schieckei* Karaman & Ruffo, 1971

**DOI:** 10.3897/BDJ.8.e53864

**Published:** 2020-07-31

**Authors:** Sabrina Lo Brutto, Davide Iaciofano

**Affiliations:** 1 Dept. SteBiCeF, University of Palermo, Palermo, Italy Dept. SteBiCeF, University of Palermo Palermo Italy

**Keywords:** Crustacean amphipods, first records, endemism, Mediterranean Sea, Levant Sea

## Abstract

**Background:**

A survey has been carried out at four Israeli rocky sites to evaluate the diversity of the amphipod fauna on various hard substrates, still scarcely monitored, as potential pabulum for amphipod crustacean species.

**New information:**

A survey of shallow rocky reefs along the Mediterranean coast of Israel recovered 28 species and integrated the Amphipoda checklist for the country ofIsrael with 12 newly-recorded species. Such renewed national list includes *Maera schieckei* Karaman & Ruffo, 1971, a rare species endemic to the Mediterranean Sea, recorded here for the first time from the southern Levant Basin. The species, described from specimens collected in the Tyrrhenian Sea in 1970, has been only recorded eight times within the whole Mediterranean Sea. A revision of the bibliography on the distribution and ecology of *M. schieckei* showed that, although mentioned only for the western Mediterranean basin by some authors, it is listed in the checklist of amphipods of the Aegean Sea and neighbouring seas and has been found in the eastern Mediterranean basin since 1978. *Maera schieckei* was rarely found in the Mediterranean, one of the most studied marine biogeographic region as concerns the amphipod fauna; and the species seems to prefer bays or gulf areas. The role of updating and monitoring faunal composition should be re-evaluated.

## Introduction

The link between the variation of biodiversity over time and the change of marine environment detected nowadays is becoming a challenge for different taxonomists' communities, which aim to re-evaluate the role of taxonomy in actual and historical collections, mainly if selectively focused on specific taxa (Coll et al. 2010, Beermann and Franke 2011, Hawkins et al. 2013, Pyke and Ehrlich 2010, Scipione 2013, De-La-Ossa-Carretero et al. 2010, Costello et al. 2010, Costello 2020). The updating of checklists (Sorbe et al. 2002, Christodoulou et al. 2013, Ferrario et al. 2017) is now becoming the prerequisite for ecological and monitoring studies, also in the light of invasive alien species monitoring (Cao et al. 2003, Servello et al. 2019).

Many habitats often represent a sink for invasive alien species, unknown species (not yet described) or rare species. Those can be the artificial (Bonifazi et al. 2018) or biogenic (Plicanti et al. 2016, Bertocci et al. 2017) hard substrates, which can significantly influence the faunal composition especially in countries, such as Israel, where sandy and muddy bottoms show a large extension along the coast. In some cases, such scattered habitats create the favourable conditions for settlement and successive establishment, facilitating the dispersal of the species also over long distances. An example was the vermetid platforms habitat whose shape and structure favoured the settlement and successive increasing density of the lessepsian mussel *Brachidontes pharaonis* (P. Fisher, 1870) (Sirna Terranova et al. 2006, Rilov et al. 2004), now widespread in the eastern and central Mediterranean (Servello et al. 2019).

In the light of that, a survey has been carried out at four Israeli rocky sites to evaluate the diversity of the amphipod fauna on variegate hard substrates (e.g. rocks, bryozoans, sponges, seaweeds, coralligenous, plastic objects etc.) as potential pabulum for an establishment of amphipod crustacean species, still scarcely monitored in scattered areas of the eastern Mediterranean Sea (Lo Brutto et al. 2016, Lubinevsky et al. 2019, Sorbe et al. 2002). In European aquatic ecosystems, including the coastal Mediterranean ones, where biodiversity is changing due to climate change and the invasion of alien species, such a ubiquitous animal group, as amphipods, plays a crucial role in detecting such changes and deserves relevant attention as fundamental tool in monitoring biodiversity (Borja et al. 2003, Lo Brutto et al. 2013).

## Materials and methods

**Figure 1.**
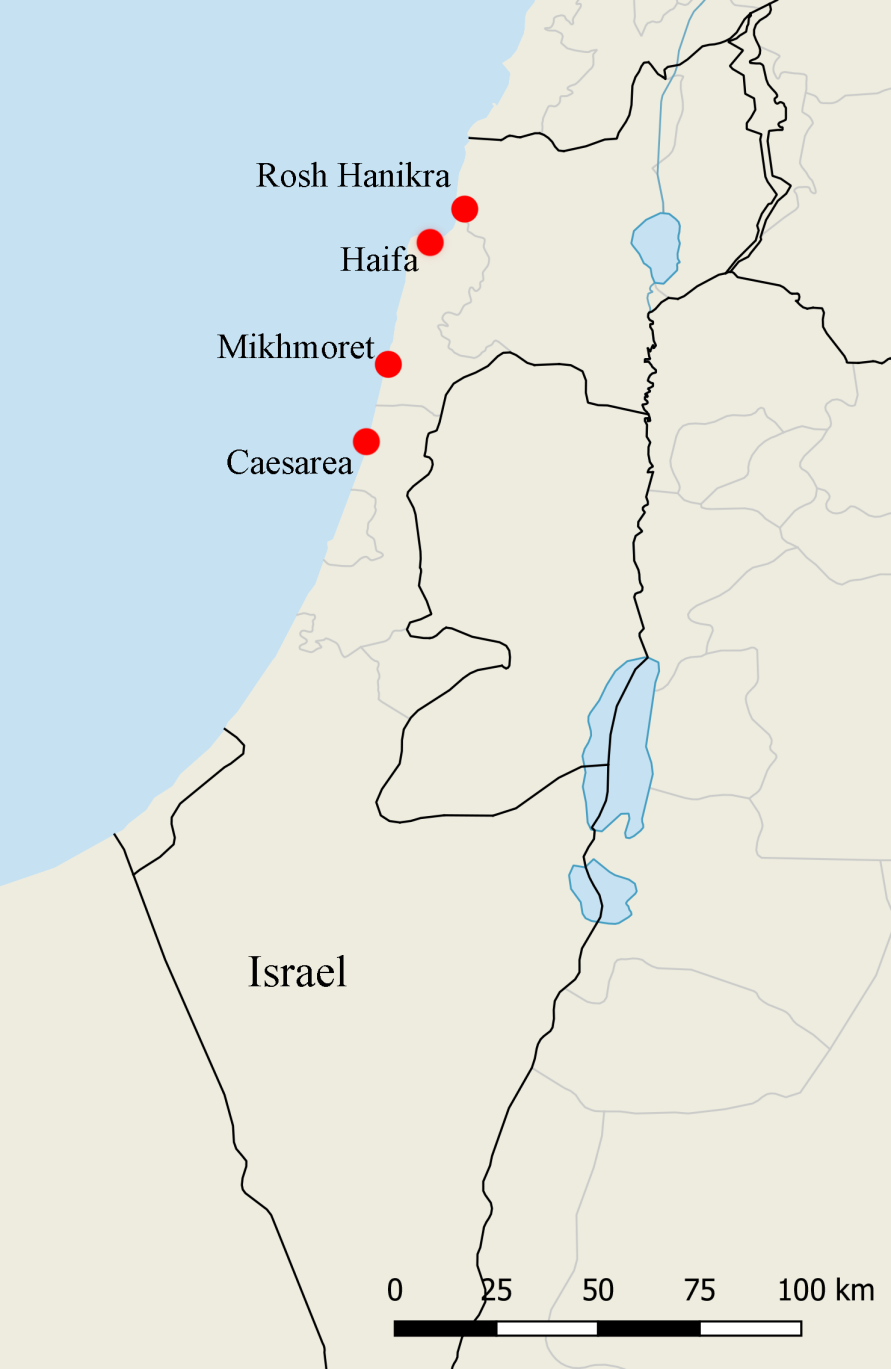
Sample sites.

Four shallow rocky reefs (intertidal - 25 m depth) off the Israeli Mediterranean coast were sampled by SCUBA diving in 2009: (Rosh Hanikra (RH) (33°04'20.35''N; 35°05'42.10''E); Haifa (HF) (32°48'47.42''N; 34°57'16.15''E); Caesarea (CS) (32°29'07.34''N; 34°53'19.93''E) and Mikhmoret (MM) (32°24'28.52''N; 34°52'02.38''E) (Fig. 1). Crustaceans, hydroids, bryozoans, sponges, macrophytes and coraligenous matrices were collected and preserved in EtOH 70%. Amphipods were examined under a stereomicroscope and dissected. Body length, from tip of rostrum to apex of telson, was measured using by ImageJ software after placement on graph paper and photographed (FINEPIX S1800, FUJIFILM); drawings were scanned and ‘inked’ using Adobe Illustrator CS5. The specimens of *Maera schieckei* are deposited at the Museum of Zoology “P. Doderlein” of the University of Palermo (MZPA), Italy and the Steinhardt Museum of Natural History (SMNH), Tel Aviv University, Israel.

### Museum collections

Natural History Museum of Verona (NHMV), Italy: 1216-1218 *Maera schieckei* one *holotypus*, a male dissected into microslides and one male and two females, from Ischia Island, central Tyrrhenian basin. Museum of the Faculty of Fisheries of Ege University (ESFM), Turkey: ESFM-MAL/2003-2 *Maera schieckei* two individuals, but one heavily damaged from the Aegean coast of Turkey and ESFM-MAL/2005-1839 *Maera schieckei* one individual from the Levantine coast of Turkey (Bakir and Katağan 2014). Museum of Zoology "P. Doderlein" of the University of Palermo (MZPA), Italy: MZPA-AMPH-0027 *Maera schieckei* seven females from the coast of Israel (this paper).

## Data resources

**Results**

A total of 3106 amphipod specimens were collected. Most specimens (88.6%) were identified to species (Table 1), the remainder to the genus level (*Ampelisca* sp., N = 27; *Caprella* sp., N = 5; *Corophium* sp., N = 10; *Melita* sp., N = 1; *Microdeutopus* sp., N = 187; *Quadrimaera* sp. N = 24) or remained unclassified (N = 27) being immature or damaged. The most abundant species were tube-dwelling: *Leptocheirus guttatus* (N = 1227), *Ericthonius brasiliensis* (N = 285), *Ampithoe ramondi* (N = 260), *Photis longicaudata* (N = 153).

The list of the 28 species (Table 1) integrates with the last checklist published for the whole Israeli coast by Sorbe et al. (2002) and adds 12 species not recorded yet up to now. Six are exclusively from the Mediterranean Sea, eight have been already recorded from the Mediterranean and the Atlantic Ocean and 14 have a wider distribution, including the Red Sea, the Indian and Pacific Oceans. Two amongst them are considered to have entered the Mediterranean Sea through the Suez Canal: *Elasmopus pectenicrus* and *Bemlos leptocheirus* (Marchini and Cardeccia 2017) (see Table 1 for details). Specimens of *Maera schieckei* collected at Rosh Hanikra and Haifa constitute the first recorded in the southern Levant Sea.

## Taxon treatments

### Maera schieckei

Karaman and Ruffo, 1971

C0AB6DEA-14F2-55D4-8A87-232DC75D970B

https://eol.org/pages/46530501

http://www.catalogueoflife.org/annual-checklist/2019/details/species/id/ea87d3953b995921e521c66e0eac83a6

http://www.marinespecies.org/amphipoda/aphia.php?p=taxdetails&id=102825

Maera schieckei described in Karaman and Ruffo 1971: p. 132, fig. 11-13

#### Materials

- **Type status:** Other material. **Occurrence:** catalogNumber: MZPA-AMPH-0027; recordedBy: S. Piraino; sex: 5 females; lifeStage: adult; **Taxon:** scientificName: *Maera schieckei* Karaman and Ruffo, 1971; order: Amphipoda; family: Maeridae; subgenus: Maera; specificEpithet: schieckei; scientificNameAuthorship: Karaman and Ruffo, 1971; **Location:** locationID: Rosh Hanikra; waterBody: Mediterranean Sea; country: Israel; verbatimCoordinateSystem: 33°04'20.35''N, 35°05'42.10''E; **Event:** eventDate: 2009 June; **Record Level:** basisOfRecord: PreservedSpecimen
- **Type status:** Other material. **Occurrence:** catalogNumber: MZPA-AMPH-0027; recordedBy: S. Piraino; sex: 2 females; lifeStage: adult; **Taxon:** scientificName: *Maera schieckei* Karaman and Ruffo, 1971; order: Amphipoda; family: Maeridae; subgenus: Maera; specificEpithet: schieckei; scientificNameAuthorship: Karaman and Ruffo, 1971; **Location:** locationID: Haifa; waterBody: Mediterranean Sea; country: Israel; verbatimCoordinateSystem: 32°48'47.42''N, 34°57'16.15''E; **Event:** eventDate: 2009 June; **Record Level:** basisOfRecord: PreservedSpecimen

#### Description

**Figure 2.**
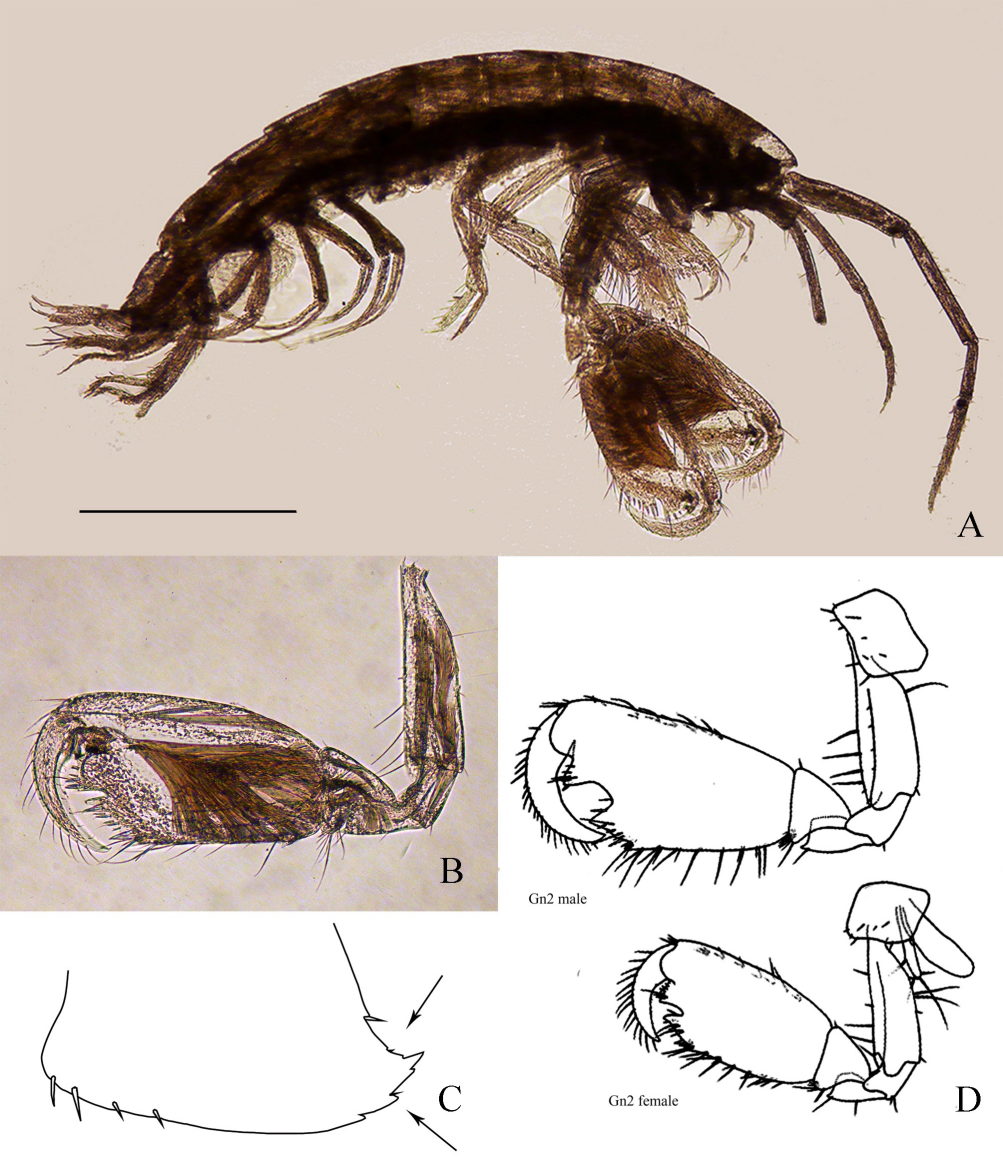
*Maera schieckei* Karaman & Ruffo, 1971; **A.** Lateral habitus; **B.** Second gnathopod female; **C.** Illustration of the third epimeral plate, with focus on the postero-distal corner teeth; **D.** Illustration of the second gnathopod male (Gn2 male) and female (Gn2 female).

**Body**: Body slender, up to 6 mm long (Fig. 2A). **Head**: Lateral cephalic lobes rounded, antennal sinus shallow; eyes subrounded. Antenna 1 length about 1/2 body, peduncle articles 1-2 subequal, article 3 shorter; flagellum with 9 articles, shorter than peduncle; accessory flagellum with 5 articles. Antenna 2, article 3 of peduncle 2× as long as broad, article 4 longer than 5, flagellum with 5-6 articles; antennal gland cone reaching tip of peduncle article 3. Mandibular palp article 1 with distal tooth, article 2 longer than 3. **Pereon**: Coxae 1-4 short, coxa 1 with anterodistal corner acutely produced. Gnathopod 1 carpus longer than propodus, propodus ovate; palm oblique, convex; dactylus with 1 anterior seta. Gnathopod 2 carpus short, propodus large, subtrapezoidal, twice as long as broad; palm with a median excavation (in male, palm slightly oblique, with deep medial excavation flanked by 2 strong teeth, defined by 2 spines and a small tooth; dactylus stout, with row of setae on anterior margin (Fig. 2B, D). Peraeopods 3-4 slender. Peraeopods 5-7 relatively slender, basis almost 2× as long as broad, posterodistal lobe present; dactylus half-length of propodus, nail short, anterior margin with 1-3 minute teeth. **Pleon**: Epimeral plates 1-2 with small posterodistal tooth. Epimeral plate 3 postero-distal corner produced with several teeth (Fig. 2C). Uropod 1 peduncle with 1 ventro-facial spine, rami subequal. Uropod 2 shorter than uropod 1, rami subequal. Uropod 3 stout, not exceeding tip of uropod 1, peduncle as long as rami; rami subequal, 1-articulate with distal spines as long as rami. Telson nearly as long as broad, deeply cleft. Telson lobes bifurcate with 2 long distal spines (of unequal length) and 3 plumose setae.

#### Distribution

**Figure 3.**
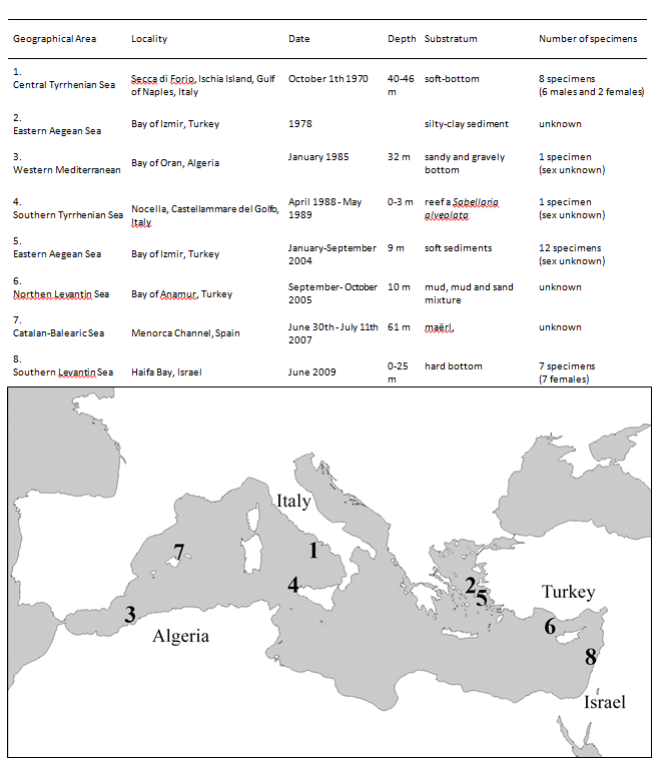
Records of *Maera schieckei* in the Mediterranean Sea pointed out in the map below. Details of the records in the Table above, from the following references: Bakalem et al. 2014, Bakir and Katağan 2014, Çinar et al. 2006, Junoy and Viéitez 2008, Karaman and Ruffo 1971, Kocataş and Katağan 1978, Lo Brutto 1991.

Mediterranean. Italy: Tyrrhenian Sea, Gulf of Naples (Karaman and Ruffo 1971). Spain: Menorca Channel (Junoy and Viéitez 2008). Algeria: Bay of Oran (Bakalem et al. 2014). Italy: Gulf of Castellammare (Lo Brutto 1991); Turkey: Bay of Izmir (Kocataş and Katağan 1978, Çinar et al. 2006); Anamur Bay (Bakir and Katağan 2014); Israel: Haifa Bay (Fig. 3). General: Mediterranean endemic.

#### Notes

Five species of *Maera* have been recorded in the Mediterranean Sea: *M. grossimana* (Montagu, 1808), *M. hirondellei* Chevreux, 1900, *M. pachytelson* Karaman & Ruffo, 1971, *M. schieckei* and *M. sodalis* Karaman & Ruffo, 1971 (accessed at http://www.marinespecies.org/amphipoda on 05-04-2020). *Maera schieckei* is distinguished from congeneric Mediterranean species by the presence of a median U-shaped excavation in the palm of the second gnathopods and several teeth on the posterodistal corner of third epimeral plate (character not always appreciable) (Fig. 2B, C, D). The genus *Maera*, erected by Leach (1814), is one of the oldest amphipod genera, which has undergone extensive revision, throughout which *Maera schieckei* has maintained its original name and taxonomic position.

The specimens described in this study corresponds to the morphology of *Maera schieckei* as described by Karaman and Ruffo 1971 and available also in Ruffo (1982).

## Discussion

In spite of their important ecological role within benthic ecosystems, hard bottom amphipods were rarely investigated on the Mediterranean coast of Israel compared with the soft-bottom ones (Lubinevsky et al. 2019, Lo Brutto et al. 2016, Sorbe et al. 2002).

Of the 28 amphipod species identified from the rocky reefs, 16 had been previously recorded (Sorbe et al. 2002) and 12 are new records for Israel (see Table 1), whereas 19 and 20 had been recorded off Cyprus and the Levantine coast of Turkey, respectively (Bakir et al. 2014, Kocataş et al. 2001 and references herein). *Bemlos leptocheirus* (Walker, 1909), occasionally recorded as Mediterranean alien species and *Synchelidium longidigitatum* (Ruffo, 1947), an endemic Mediterranean species, are the first records for the whole Levantine Sea.

Currently, the Mediterranean Sea suffers a high anthropogenic impact due to warming water, internal and external boat traffic and pollution (Galil 2000, Occhipinti-Ambrogi 2007). The effects of these activities can be assessed by the spread of some species, for which the hard substratum can be a point of the pathway. Several studies focused on the spread of alien species (Marchini and Cardeccia 2017, Servello et al. 2019, Ulman et al. 2017) and do not often consider the spread of the autochthonous species as an additional significant signal of changing.

The value of the Mediterranean basin in relation to its role as a hotspot of endemisms can be enhanced also by the capture of rare species. *Maera schieckei* is here identified for the first time along the Israeli coast and it is the most south-eastern record of the species in the Mediterranean Sea (Fig. 2). This rare Mediterranean endemic species has only been collected from a few locations, with a low number of specimens found only four times in the western basin and three times in the eastern basin (Fig. 3). The zoogeography of *M. schieckei* is scarcely known and, in some cases, inaccurate. Though Christodoulou et al. (2013) recently reported the species only present in the western Mediterranean basin and it was not listed in the check-list of the north Aegean Sea (Stefanidou and Voultsiadou-Koukoura 1995) and Israel (Sorbe et al. 2002), this species has been identified in the eastern Mediterranean basin since 1978.

Even if this species occurred within a wide geographical range (approximatively all over the Mediterranean basin), in a wide habitat specificity (different types of substrate), it was recorded only in bay or gulf areas, often polluted and degraded areas due to the presence of commercial harbours (see references in Fig. 3); and it remains uncertain if a small-sized crustacean can spread for hundreds of kilometres along the Mediterranean basin and be recorded only few times. Studies on its life history needs to better understand dispersal and establishment. Data suggest it to be an opportunistic species. It is noteworthy to mention Ferrario et al. (2017) who evaluated the role of harbours in spreading non-native species; they can be probably significant in driving the range expansion of autochthonous species as well.

Its small populations make the species being attributed to a rarity with wide geographical range and low frequency of occurrence (Rabinowitz 1981) and the data deficiency collocating it as species Not Evaluated (NE), according to International Union for Conservation of Nature (IUCN) 2001; in this case, the IUCN recommends to give it the same degree of attention as threatened taxa, at least until the status can be assessed.

A recent estimate fixes the number of benthic amphipod species in the Mediterranean basin at 449 (Coll et al. 2010). Yet, there is no doubt that this number is an underestimate and that we lack information for wide swathes of the region as research efforts vary greatly along the coasts of the Mediterranean and even amongst particular habitats within well-studied areas (Lo Brutto et al. 2019, Curatolo et al. 2013). A cursory examination of recent publications reveals the magnitude of the gaps. The number of species and genera new to science, some described from material collected in well-studied areas, confirm that the actual number of Mediterranean amphipods is a function of search effort and taxonomic expertise (Coleman 2015). For instance, a study along the Algerian coast listed 33 new records (Bakalem et al. 2014) and, even along the Spanish coast, a study of the shallow soft bottom fauna listed five new records and 14 second records (De-La-Ossa-Carretero et al. 2010). Records of native ‘rare’ species, whose role in the ecosystem and importance in bioassessment is still debated (Cao et al. 2003), accrue as well (Scipione 2013). The Marine Science Framework Directive, with its descriptors for 'good environmental status' (Review of the Commission Decision 2010/477/EU concerning MSFD criteria), opens new perspectives to monitor, manage and protect the marine environment. The three criteria for the assessment of any species are distribution, population size and population condition and special attention is called to the “*integrated understanding of the distribution, extent and condition of their habitats … to make sure that there is a sufficiently large habitat to maintain its population, taking into consideration any threat of deterioration or loss of such habitats*.” During an era of intensification of anthropogenic activities which drive complex and fundamental changes in the Mediterranean Sea (European Environment Agency, EEA 2015), it is important to strengthen and augment the study of the faunal diversity of the Mediterranean Sea – a sea notable for its endemisms.

## Supplementary Material

XML Treatment for Maera schieckei

## Figures and Tables

**Table 1.** List of species recorded per site, with their abundance, biogeographical distribution^1^ and record along the Levantine coast of Turkey (Bakir et al. 2014) and Cyprus (Kocataş et al. 2001). AO= Atlantic Ocean; Med= Mediterranean Sea; BS= Black Sea; RS= Red Sea; IO= Indian Ocean; PO= Pacific Ocean; IPO=Indo-Pacific Ocean; *First record in Israeli waters herein presented; ^1^References from which distribution has been inferred: Ruffo 1982, Ruffo 1989, Ruffo 1993, Ruffo 1998, Bakir et al. 2014 and Christodoulou et al. 2013.

Species	Rosh Hanikra	Haifa	Mikhmoret	Caesarea	Distribution^1^	Turkey	Cyprus
*Apolochus neapolitanus* (Della Valle, 1893)*			2	3	AO-Med- IPO	Yes	No
*Ampithoe ramondi* Audouin, 1826	1		213	46	AO-Med-BS-RS-IO	Yes	Yes
*Ampithoe riedli* Krapp-Schickel, 1968*				18	Med	Yes	Yes
*Leptocheirus guttatus* (Grube, 1864)*	72	60	1052	43	AO-Med	Yes	Yes
*Bemlos leptocheirus* (Walker, 1909)*			21	18	Med-IO	No	No
*Caprella equilibra* Say, 1818				12	Cosmopolitan	No	Yes
*Phtisica marina* Slabber, 1769		3			AO- Med-PO	Yes	Yes
*Colomastix pusilla* Grube, 1861		1			Cosmopolitan	Yes	Yes
*Dexamine spinosa* (Montagu, 1813)	4	22			AO- Med	Yes	Yes
*Tritaeta gibbosa* (Spence Bate, 1862)	34				AO- Med	No	Yes
Protohyale (Boreohyale) camptonyx (Heller, 1866)*		7		67	AO-Med-RS	Yes	Yes
*Ericthonius brasiliensis* (Dana, 1853)*			154	131	AO-Med-IO	No	Yes
*Coxischyrocerus inexpectatus* (Ruffo, 1959)				2	Med	Yes	No
Leucothoe cf. spinicarpa (Abildgaard, 1789)	2			2	Cosmopolitan	Yes	Yes
*Lysianassa caesarea* Ruffo, 1987	3	23	39	26	Med	Yes	Yes
*Elasmopus pectenicrus* (Spence Bate, 1862)				34	AO-Med -RS-IO-PO	Yes	No
*Elasmopus pocillimanus* (Spence Bate, 1862)				4	AO-RS-IO-Med	No	Yes
*Maera grossimana* (Montagu, 1808)*			23		AO-Med-BS	Yes	Yes
*Maera schieckei* Karaman & Ruffo, 1971*	5	2			Med	Yes	No
*Quadrimaera inaequipes* (A. Costa, 1851)*	68	47	32	154	Cosmopolitan	Yes	Yes
*Perioculodes longimanus* (Bate & Westwood, 1868)				10	AO-RS-IO-Med	Yes	Yes
*Synchelidium longidigitatum* Ruffo, 1947*	12	32	18		Med	No	No
*Megamphopus brevidactylus* Myers, 1976				4	Med	No	No
*Photis longicaudata* (Spence Bate & Westwood, 1862)	1		118	34	AO- Med-IO	Yes	No
*Metaphoxus simplex* (Spence Bate, 1857)	1	8	10	15	AO- Med	Yes	No
*Podocerus variegatus* Leach, 1814			1	21	AO- Med	Yes	Yes
*Stenothoe tergestina* (Nebeski, 1880)*				7	AO- Med	Yes	Yes
*Stenothoe dollfusi* Chevreux, 1887*	5				AO- Med	No	Yes
